# Genetic evidence for conserved non-coding element function across species–the ears have it

**DOI:** 10.3389/fphys.2014.00007

**Published:** 2014-01-21

**Authors:** Eric E. Turner, Timothy C. Cox

**Affiliations:** ^1^Center for Integrative Brain Research, Seattle Children's Research InstituteSeattle, WA, USA; ^2^Center on Human Development and Disability, University of WashingtonSeattle, WA, USA; ^3^Department of Psychiatry and Behavioral Sciences, University of WashingtonSeattle, WA, USA; ^4^Department of Pediatrics (Craniofacial Medicine), University of WashingtonSeattle, WA, USA; ^5^Department of Anatomy and Developmental Biology, Monash UniversityClayton, VIC, Australia; ^6^Center for Developmental Biology and Regenerative Medicine, Seattle Children's Research InstituteSeattle, WA, USA

**Keywords:** Hmx1, pinna, ear development, craniofacial morphogenesis, enhancer, oculoauricular syndrome

## Abstract

Comparison of genomic sequences from diverse vertebrate species has revealed numerous highly conserved regions that do not appear to encode proteins or functional RNAs. Often these “conserved non-coding elements,” or CNEs, can direct gene expression to specific tissues in transgenic models, demonstrating they have regulatory function. CNEs are frequently found near “developmental” genes, particularly transcription factors, implying that these elements have essential regulatory roles in development. However, actual examples demonstrating CNE regulatory functions across species have been few, and recent loss-of-function studies of several CNEs in mice have shown relatively minor effects. In this Perspectives article, we discuss new findings in “fancy” rats and Highland cattle demonstrating that function of a CNE near the *Hmx1* gene is crucial for normal external ear development and when disrupted can mimic loss-of function Hmx1 coding mutations in mice and humans. These findings provide important support for conserved developmental roles of CNEs in divergent species, and reinforce the concept that CNEs should be examined systematically in the ongoing search for genetic causes of human developmental disorders in the era of genome-scale sequencing.

## The concept of conserved non-coding elements

In the past decade the availability of the genomic sequences of diverse animal species has led to the recognition of a high degree of sequence conservation outside protein coding regions, particularly across vertebrate genomes. While some of this sequence conservation may be accounted for by non-coding functional RNA, most of the conserved regions are believed to have *cis*-regulatory function. Genomic regions that can be recognized by the alignment of distantly related vertebrate species, such as fish, rodents, and humans, have been termed “conserved non-coding elements” or CNEs (Nelson and Wardle, 2013). The concept of a CNE is related to the term “ultraconserved element” (UCE), although this latter designation refers specifically to sequences of at least 200 bp with 100% conservation between rodent and human genomes (Sandelin et al., 2004), which is not an essential determinant of function (Visel et al., 2008). CNEs are also conceptually related to “cis-regulatory modules,” or CRMs, which are defined as regulatory elements that interact with transcription factors to determine tissue-specific gene expression (Howard and Davidson, 2004). However, the recognizable conservation of an extended sequence across species is not a defining feature of CRMs.

Indirect evidence for the regulatory function of CNEs is supplied by the frequent occurrence of these sequences near developmental regulatory genes, particularly transcription factors (Sandelin et al., 2004). This observation reinforces the impression that CNEs regulate core programs of gene expression related to cell identity and morphogenesis which must be solved by all vertebrates. More direct evidence for enhancer function has been obtained from experiments in which CNEs have been used to drive tissue-specific gene expression in zebrafish (Woolfe et al., 2005) and in mice (Pennacchio et al., 2006). Similarly, two reports to date describing efforts to delete numerous CNEs with known enhancer function in mice have shown surprisingly minor phenotypic effects (Ahituv et al., 2007; Attanasio et al., 2013). This low yield in targeted or “reverse” genetic experiments demonstrates that forward genetic strategies have an important role in determining CNE function.

## CNEs as sites of disease causing mutations: promises unfulfilled

If CNEs have conserved regulatory functions, mutations in these sequences should present with related phenotypes in diverse species. However, support for this prediction to date has been sparse. One of the few clear examples of a conserved CNE phenotype relates to a long-range enhancer regulating expression of the morphogen Shh in the developing limb, which is the site of causative alleles in preaxial polydactyly in humans (Lettice and Hill, 2005), and distal limb defects in the mouse insertional mutant sasquatch (ssq, Sharpe et al., 1999). The function of the relevant enhancer, residing ~1Mb from the coding gene, has been confirmed by targeted mutagenesis (Sagai et al., 2005). The causative alleles for a small number of other human genetic disorders have mapped outside coding sequence (Amiel et al., 2010), but in most cases the gene regulatory mechanisms involved are unclear, and cross-species demonstration of CNE function has not been obtained. This is partly because the assignment of function to sequence variation found in CNEs is more difficult than that found in coding sequence, since the “transcriptional genetic code” that governs transcription factor binding to enhancer sequences is less well-understood and more complex than the translational genetic code governing coding sequence.

## Hmx1: taking the bull by the horns (or ears)

**Figure 1:**
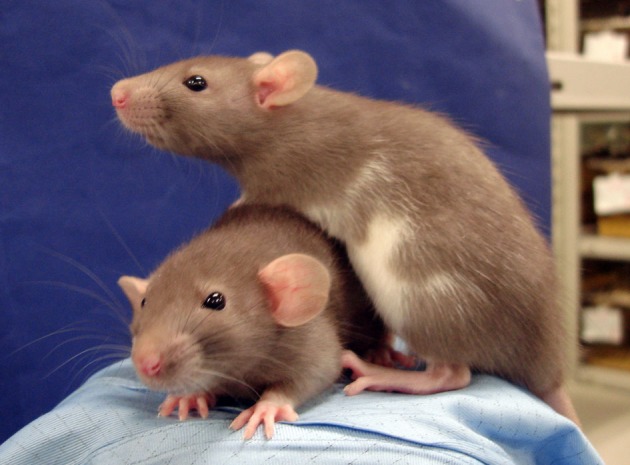
**Rats exhibiting normal (top) and Dumbo (bottom) ear phenotypes Kuramoto et al. (2010)**.

Two recent reports of mutations affecting development of the external ear, one in “fancy” rats kept by amateur breeders in the United States, and the other in Highland cattle bred in Switzerland, provide exciting new evidence for disruption of CNE function as a mechanism underlying developmental disorders (Quina et al., 2012a; Koch et al., 2013). The rat “dumbo” (*dmbo*) mutation is a recessive trait causing ventral displacement and rotation of the external ear (pinna), leading to protruding ears akin to the cartoon elephant that gives the strain its name (Figure 1, Kuramoto et al., 2010). The origin of the dumbo phenotype is obscure, but it was probably first identified by hobbyist breeders in the western United States, and it is considered a desirable trait in fancy rats. The “crop ear” trait in Highland cattle shows partially dominant inheritance of a moderately to severely truncated (or cropped) ear deformity, which may vary according to gene dosage and genetic background (Scheider et al., 1994). Crop-eared Highland cattle are found in herds in Europe, North America, and Australia. In some regions the breeding of animals with severe crop ear defects is discouraged, in others it is ignored, since it has no other known effect on the health of the affected animal.

**Figure 2:**
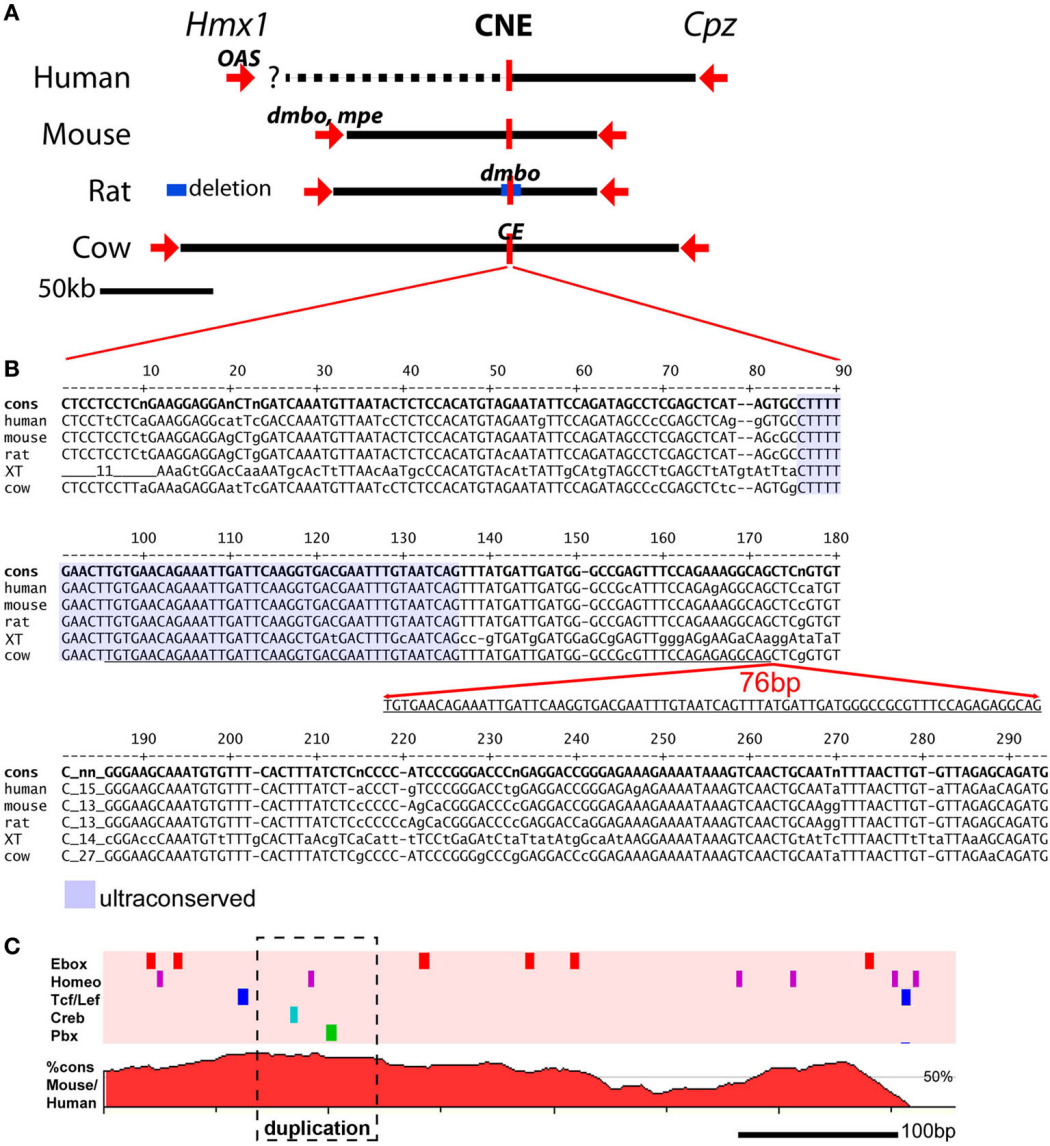
**Genomic location of mutations affecting pinna development at the Hmx1 locus. (A)** The chromosomal relationship between the *Hmx1* gene (and in some species not shown, two related *Hmx* genes), a distal *Hmx1* CNE, and the neighboring gene encoding carboxypeptidase Z (*Cpz*) is conserved in all known vertebrate genomes (Quina et al., 2012b). The human OAS allele is a 26 bp deletion in the coding region of *Hmx1*, resulting in a frameshift, which is likely to be a null allele (Schorderet et al., 2008). The mouse *dmbo* and *mpe* alleles also map to the *Hmx1* coding region, and are also likely to be null alleles (Jiang et al., 2002; Munroe et al., 2009). In contrast, the rat *dmbo* allele is a 5777 bp deletion encompassing the CNE (Chieffo et al., 1997), and the bovine *crop ear* (CE) allele is a 76 bp duplication within the CNE (Koch et al., 2013). **(B)** Alignment of the *Hmx1* distal CNE sequence from the human, mouse, rat, *xenopus tropicalis* and cow genomes. Conserved bases are shown in upper case. Alignment of the *x. tropicalis* sequence with the mammalian genomes identifies a core “ultraconserved” sequence (shaded area) that can be identified in all vertebrate genomes for which data are available (Quina et al., 2012b). The 76 bp CE allele consists of a duplication within this core element. The genomic coordinates of the sequences shown are as follows: human, GRCh37/hg19 chr4:8,702,169-8,702,467; mouse, GRCm38/mm10, chr5:35,466,174-35,466,471; rat, Baylor 3.4/rn4, chr14:80,916,333-80,916,631; xenopus tropicalis, JGI 4.2/xenTro3, GL173219:238,403-238,694; cow, Baylor Btau_4.6.1/bosTau7, chr6:120,904,093-120,904,404. **(C)** Conserved transcription factor binding sites (top) and VISTA plot (bottom) of the Hmx1 CNE. Five TF binding sites for TF families of known developmental significance occur in this region; only those sites conserved across seven mammalian species are shown. In a DNA sequence of this length, the random expected occurrence of Ebox sites is two, of homeobox sites is two, and of the other sites is zero. VISTA plot shows percent homology between human and mouse genomes. The conserved transcription factor search was performed using Mulan: http://mulan.dcode.org/.

Genetic mapping of the rat *dmbo* and bovine *crop ear* alleles converged on chromosomal locations that encompass the *Hmx1* gene, a homeodomain transcription factor (Figure 2A). Coding mutations at the *Hmx1* locus are known to account for two mouse ear variants, one also called “dumbo,” (*dmbo*), and one called “misplaced ears” (*mpe*, Munroe et al., 2009). The mouse *dmbo* and *mpe* alleles consist of a nonsense mutation and an 8 bp coding region deletion, respectively, and are likely to be null alleles. In humans, a coding variant of the *HMX1* gene causes a recessive disorder called oculoauricular syndrome (OAS), characterized by deformities of the pinna and also variable eye defects (Schorderet et al., 2008; Vaclavik et al., 2011). The human *HMX1* allele associated with OAS consists of a 26 bp deletion in the coding region, resulting in a frameshift, which is also likely to be a null allele.

Remarkably, the rat *dmbo* and bovine *crop ear* mutations do not affect the Hmx1 coding region. Instead, resequencing of the chromosomal regions containing *Hmx1* in these species revealed mutations far downstream from the *Hmx1* transcription unit (Figure 2A). In the rat, the *dmbo* allele consists of a 5777 bp deletion residing ~80 Mb downstream of the *Hmx1* transcription unit. A CNE of ~300 bp within this region exhibits very high identity (85–98%) between all mammalian species, and exhibits a core of conserved sequence that is retained in reptiles, fish and amphibians (Figure 2B). The bovine crop ear allele consists of a 76 bp duplication within the most highly conserved part of this CNE.

Supporting evidence for tissue-specific enhancer function within the rat *dmbo* deletion region, likely conferred by this CNE, has been provided by studies of developmental gene expression in dumbo rat embryos (Quina et al., 2012a). Embryonic dumbo rats exhibit loss of Hmx1 protein expression specifically in the craniofacial mesenchyme which contributes to the pinna. In contrast, expression is normal in the cranial sensory nervous system, where Hmx1 also has an important developmental role (Quina et al., 2012b). The Hmx1 CNE is rich in predicted binding sites for transcription factor classes known to be important in regulating developmental processes (Figure 2C). Unfortunately, the prediction of enhancer function from sequence data is not reliable enough to infer whether the additional sites contained in the *crop ear* duplication will increase enhancer function or disrupt it.

The ear phenotype of dumbo rats closely resembles that seen in the mouse loss-of-function alleles, with pinnae that are slightly dysmorphic and ventrally displaced and rotated, giving the appearance of larger “floppy” ears. The bovine *crop ear* phenotype in contrast is a marked pinna deficiency. These differences may reflect apposing effects of the mutations: i.e., loss of Hmx1 expression in rats and increased expression in cattle. With respect to this, it is also interesting to note that the bovine *crop ear* trait is partially dominant, whilst the rodent *Hmx1* alleles, including the rat *dmbo* CNE deletion, are recessive. It will be interesting to see whether these distinct modes of transmission result from the specific effect of the *crop ear* duplication event on Hmx1 enhancer function, or interspecies differences in the effects *Hmx1* gene dosage on pinna development. Regardless, these cross-species examples reinforce the role of CNEs in conserved developmental processes and the notion that they can be a cause of developmental disorders.

## Future directions

Extensive evidence for conserved CNE enhancer function probably exists in the gene pool of the diverse vertebrates for which we now have nearly complete genomic sequence. The similarity of the phenotypes seen in *Hmx1* coding and CNE mutants in mice, rats, cattle and humans indicate that CNE mutant phenotypes will not necessarily be subtle or polygenic, but instead may show Mendelian inheritance with high penetrance, and may phenocopy at least some of the features associated with null mutations in the genes they regulate. Although work in this area has just begun, three general strategies can advance the identification of functional mutations in CNEs that underlie human disease.

First, functional variants in CNEs will escape detection using the current methods of genomic analysis, particularly whole exome sequencing and transcriptome sequencing, since CNEs reside in introns and intergenic sequences. The CNEs identified in the human genome should therefore be systematically added to whole exome analysis of human genetic disorders, and examined for copy number variation, in order to appreciate the full range of sequence diversity in these regions that may cause disease phenotypes. The recognition of functional changes in CNEs would be greatly facilitated by a complete catalog of the genetic diversity in these loci, in the human population as well as across species.

Second, even when sequence variants in CNEs are identified, our ability to decode changes in CNE function based on sequence variation is still quite limited, especially for single nucleotide polymorphisms. General evidence for the enhancer function of a CNE, although not its specific regulatory role, can be derived from the analysis of local chromatin states by chromatin immunoprecipitation of modified histones and DNA methylation analysis (Schones and Zhao, 2008). Such genome-wide analyses of the human and mouse chromatin landscapes in several cell types are now accessible in searchable databases (Ernst et al., 2011; Shen et al., 2012). However, the general assignment of regulatory importance to a CNE-containing region does not determine the significance of any subtle genetic variants found within it. In principle this determination should be based on decoding the transcription factor binding sites within a CNE, thus allowing the recognition of important regulatory mutations, even those resulting from single-nucleotide changes. However, the flexible and combinatorial nature of transcription factor binding, and the large number of factors encoded by vertebrate genomes, make this an intrinsically difficult problem (Meireles-Filho and Stark, 2009), but one which is becoming tractable with advances in the systematic analysis of transcription factor binding sites (Jolma et al., 2013).

Finally, the definitive functional test of a disease-associated CNE can only be performed by demonstrating gain or loss of regulatory function in an appropriate *in vivo* vertebrate model, such as transgenic mice (Pennacchio et al., 2006; Ahituv et al., 2007) or zebrafish (Fisher et al., 2006), or the electroporation of enhancer-linked reporters in chick embryos (Simoes-Costa and Bronner, 2013). These methods are laborious and of moderate throughput, and thus will usually be applied to candidate CNEs that have already met some of the other criteria for probable significance. However, when successful, enhancer analysis in model species can reveal not only evidence for a disease-causing mechanism, but also the fundamental relationship between gene regulation and species-specific traits (Wittkopp and Kalay, 2012). For instance, in one effort at cross-species functional CNE analysis, replacement of a limb-specific enhancer in the Prx1 gene of the mouse with the orthologous sequence from a species of bat was shown to result in forelimb elongation (Cretekos et al., 2008). The key to an efficient strategy for definitive proof of CNE function in model systems will be to leverage as much as possible the available clues derived from forward genetic analysis of spontaneously occurring variants, such as the Hmx1-linked ear malformation syndromes, and also the gene regulatory framework provided by less-specific but more comprehensive genomic and epigenomic analysis.

### Conflict of interest statement

The authors declare that the research was conducted in the absence of any commercial or financial relationships that could be construed as a potential conflict of interest.

## Glossary

**Conserved non-coding element (CNE)**.: A genomic region defined by sequence conservation between species, without known or predicted protein coding function. Such sequences are hypothesized to have regulatory functions.
**Cis-regulatory module (CRM)**.: A genomic region defined by function that contains recognition sites for transcription factors and affects the regulation of a gene. CRMs may be CNEs but are not necessarily recognizable from sequence conservation alone.
**Enhancer**.: A region of chromosomal DNA that can mediate the activation of the tissue-specific expression of a gene. Most identified enhancers reside within introns or in the intergenic space between a gene and its chromosomal neighbors. However, examples exist of enhancers located at megabase distances from their regulatory targets.
**H6 family homeobox 1 (Hmx1, HMX1/NKX5.3)**.: A homeodomain gene/protein with expression in the developing external ear, eye, and peripheral sensory ganglia.
**Oculoauricular syndrome (OAS)**.: A human congenital syndrome involving malformation of the eye and external ear, one form of which has been linked to *HMX1*.
**Ultraconserved element (UCE)**.: A CNE with perfect or near-perfect interspecies conservation.

## References

[B1] Ahituv N. Zhu Y. Visel A. Holt A. Afzal V. Pennacchio L. A. (2007). Deletion of ultraconserved elements yields viable mice. PLoS Biol. 5:e234 10.1371/journal.pbio.0050234 17803355 PMC1964772

[B2] Amiel J. Benko S. Gordon C. T. Lyonnet S. (2010). Disruption of long-distance highly conserved noncoding elements in neurocristopathies. Ann. N.Y. Acad. Sci. 1214, 34–46 10.1111/j.1749-6632.2010.05878.x 21175683

[B3] Attanasio C. Nord A. S. Zhu Y. Blow M. J. Li Z. Liberton D. K. (2013). Fine tuning of craniofacial morphology by distant-acting enhancers. Science 342, 1241006 10.1126/science.1241006 24159046 PMC3991470

[B4] Chieffo C. Garvey N. Gong W. Roe B. Zhang G. Silver L. (1997). Isolation and characterization of a gene from the DiGeorge chromosomal region homologous to the mouse Tbx1 gene. Genomics 43, 267–277 10.1006/geno.1997.4829 9268629

[B5] Cretekos C. J. Wang Y. Green E. D. Martin J. F. Rasweiler J. J. T. Behringer R. R. (2008). Regulatory divergence modifies limb length between mammals. Genes Dev. 22, 141–151 10.1101/gad.1620408 18198333 PMC2192750

[B6] Ernst J. Kheradpour P. Mikkelsen T. S. Shoresh N. Ward L. D. Epstein C. B. (2011). Mapping and analysis of chromatin state dynamics in nine human cell types. Nature 473, 43–49 10.1038/nature09906 21441907 PMC3088773

[B7] Fisher S. Grice E. A. Vinton R. M. Bessling S. L. Urasaki A. Kawakami K. (2006). Evaluating the biological relevance of putative enhancers using Tol2 transposon-mediated transgenesis in zebrafish. Nat. Protoc. 1, 1297–1305 10.1038/nprot.2006.230 17406414

[B8] Howard M. L. Davidson E. H. (2004). cis-Regulatory control circuits in development. Dev. Biol. 271, 109–118 10.1016/j.ydbio.2004.03.031 15196954

[B9] Jiang X. Iseki S. Maxson R. E. Sucov H. M. Morriss-Kay G. M. (2002). Tissue origins and interactions in the mammalian skull vault. Dev. Biol. 241, 106–116 10.1006/dbio.2001.0487 11784098

[B10] Jolma A. Yan J. Whitington T. Toivonen J. Nitta K. R. Rastas P. (2013). DNA-binding specificities of human transcription factors. Cell 152, 327–339 10.1016/j.cell.2012.12.009 23332764

[B11] Koch C. T. Bruggmann R. Tetens J. Drogemuller C. (2013). A Non-coding genomic duplication at the HMX1 locus is associated with crop ears in highland cattle. PLoS ONE 8:e77841 10.1371/journal.pone.0077841 24194898 PMC3806818

[B12] Kuramoto T. Yokoe M. Yagasaki K. Kawaguchi T. Kumafuji K. Serikawa T. (2010). Genetic analyses of fancy rat-derived mutations. Exp. Anim. 59, 147–155 10.1538/expanim.59.147 20484848

[B13] Lettice L. A. Hill R. E. (2005). Preaxial polydactyly: a model for defective long-range regulation in congenital abnormalities. Curr. Opin. Genet. Dev. 15, 294–300 10.1016/j.gde.2005.04.002 15917205

[B14] Meireles-Filho A. C. Stark A. (2009). Comparative genomics of gene regulation-conservation and divergence of cis-regulatory information. Curr. Opin. Genet. Dev. 19, 565–570 10.1016/j.gde.2009.10.006 19913403

[B15] Munroe R. J. Prabhu V. Acland G. M. Johnson K. R. Harris B. S. O'Brien T. P. (2009). Mouse H6 Homeobox 1 (Hmx1) mutations cause cranial abnormalities and reduced body mass. BMC Dev. Biol. 9:27 10.1186/1471-213X-9-27 19379485 PMC2676275

[B16] Nelson A. C. Wardle F. C. (2013). Conserved non-coding elements and cis regulation: actions speak louder than words. Development 140, 1385–1395 10.1242/dev.084459 23482485

[B17] Pennacchio L. A. Ahituv N. Moses A. M. Prabhakar S. Nobrega M. A. Shoukry M. (2006). *In vivo* enhancer analysis of human conserved non-coding sequences. Nature 444, 499–502 10.1038/nature05295 17086198

[B18] Quina L. A. Kuramoto T. Luquetti D. V. Cox T. C. Serikawa T. Turner E. E. (2012a). Deletion of a conserved regulatory element required for Hmx1 expression in craniofacial mesenchyme in the dumbo rat: a newly identified cause of congenital ear malformation. Dis. Model. Mech. 5, 812–822 10.1242/dmm.009910 22736458 PMC3484864

[B19] Quina L. A. Tempest L. Hsu Y. W. Cox T. C. Turner E. E. (2012b). Hmx1 is required for the normal development of somatosensory neurons in the geniculate ganglion. Dev. Biol. 365, 152–163 10.1016/j.ydbio.2012.02.022 22586713 PMC3710741

[B20] Sagai T. Hosoya M. Mizushina Y. Tamura M. Shiroishi T. (2005). Elimination of a long-range cis-regulatory module causes complete loss of limb-specific Shh expression and truncation of the mouse limb. Development 132, 797–803 10.1242/dev.01613 15677727

[B21] Sandelin A. Bailey P. Bruce S. Engstrom P. G. Klos J. M. Wasserman W. W. (2004). Arrays of ultraconserved non-coding regions span the loci of key developmental genes in vertebrate genomes. BMC Genomics 5:99 10.1186/1471-2164-5-99 15613238 PMC544600

[B22] Scheider A. Schmidt P. Distl O. (1994). [Inheritance of notched ears in Highland cattle]. Berl. Munch. Tierarztl. Wochenschr. 107, 348–352 7802624

[B23] Schones D. E. Zhao K. (2008). Genome-wide approaches to studying chromatin modifications. Nat. Rev. Genet. 9, 179–191 10.1038/nrg2270 18250624 PMC10882563

[B24] Schorderet D. F. Nichini O. Boisset G. Polok B. Tiab L. Mayeur H. (2008). Mutation in the human homeobox gene NKX5-3 causes an oculo-auricular syndrome. Am. J. Hum. Genet. 82, 1178–1184 10.1016/j.ajhg.2008.03.007 18423520 PMC2427260

[B25] Sharpe J. Lettice L. Hecksher-Sorensen J. Fox M. Hill R. Krumlauf R. (1999). Identification of sonic hedgehog as a candidate gene responsible for the polydactylous mouse mutant Sasquatch. Curr. Biol. 9, 97–100 10.1016/S0960-9822(99)80022-0 10021368

[B26] Shen Y. Yue F. McCleary D. F. Ye Z. Edsall L. Kuan S. (2012). A map of the cis-regulatory sequences in the mouse genome. Nature 488, 116–120 10.1038/nature11243 22763441 PMC4041622

[B27] Simoes-Costa M. Bronner M. E. (2013). Insights into neural crest development and evolution from genomic analysis. Genome Res. 23, 1069–1080 10.1101/gr.157586.113 23817048 PMC3698500

[B28] Vaclavik V. Schorderet D. F. Borruat F. X. Munier F. L. (2011). Retinal dystrophy in the oculo-auricular syndrome due to HMX1 mutation. Ophthalmic Genet. 32, 114–117 10.3109/13816810.2011.562955 21417677

[B29] Visel A. Prabhakar S. Akiyama J. A. Shoukry M. Lewis K. D. Holt A. (2008). Ultraconservation identifies a small subset of extremely constrained developmental enhancers. Nat. Genet. 40, 158–160 10.1038/ng.2007.55 18176564 PMC2647775

[B30] Wittkopp P. J. Kalay G. (2012). Cis-regulatory elements: molecular mechanisms and evolutionary processes underlying divergence. Nat. Rev. Genet. 13, 59–69 10.1038/nrg3095 22143240

[B31] Woolfe A. Goodson M. Goode D. K. Snell P. McEwen G. K. Vavouri T. (2005). Highly conserved non-coding sequences are associated with vertebrate development. PLoS Biol. 3:e7 10.1371/journal.pbio.0030007 15630479 PMC526512

